# Commentary on: “Implementing a Mediterranean-Style Diet Outside the Mediterranean Region”

**DOI:** 10.1007/s11883-018-0744-8

**Published:** 2018-07-09

**Authors:** Markos Klonizakis, Ahmad Alkhatib, Geoff Middleton

**Affiliations:** 1 Centre for Sport and Exercise Science, Sheffield Hallam University, Sheffield, S10 2BP UK; 2 Dasman Diabetes Institute, P.O. Box 1180, 15462 Dasman, Kuwait; 3 School of Sport and Exercise Science, University of Lincoln, Brayford Campus, Lincoln, Lincolnshire LN6 7TS UK

**Keywords:** Mediterranean diet, Randomised controlled trial, Commentary

Dear Editor,

As a team, which is probably the first to have purposely demonstrated the cardioprotective effectiveness of implementing a Mediterranean diet (MD) in the UK, we welcome an enthusiastic article promoting the MD in another non-Mediterranean country. However, to successfully raise awareness and convince policy-makers to alleviate any implementation fears, articles investigating this area need to be well-researched and have critical attention to the feasibility of implementing this particular diet into ‘westerners’ diets. This trait was missing from the article written by Murphy & Parletta for a previous edition of the journal [1].

Indeed, the endeavour of a number of researchers on this very topic was ignored. Our UK experience has shown promising effectiveness of MD in English cohorts, particularly in enhancing age-related and post-menopausal-related vascular and cardiorespiratory functions [2–4]. We were also the first to describe the barriers and facilitators to implementing such diet in non-Mediterranean populations for researchers to take forward when they design their studies [5]. We have further extended this advice, by highlighting the cardiometabolic protective mechanisms associated with each MD component and associated nutraceutical, as a result of adopting MD in order to prevent and manage metabolic diseases [6, 7].

Therefore, we feel strongly that our work should have been considered [2–4], particularly when the authors eluded to the RCT trials with explicit outcomes (page 3, initial paragraph), as it describes work-based on MD in conjunction with other lifestyle elements, which is a format most likely to be implemented in a wider scale intervention, if MD is adopted as a preferred dietary lifestyle component.

In view with this omission, it is worth noting that our findings are in disagreement with the authors’ opinion of MD being a ‘low-cost’ diet, when adopted in in a non-Mediterranean ‘western’ country [5]. Our participants highlighted cost, time to prepare and taste preference (especially for those who are not used to consuming olive oil) as implementation barriers [5].

Another key element of their work was the emphasis placed on the high adherence rate that they achieved 6-month trial. Interestingly, we achieved a similar adherence rate (in excess of 90%), despite having less contact with the participants—we also reported longer-term microvascular benefits (12 vs. 6 months). Our pragmatic approach was based on more initial frequent contact in the first 2 months and less frequent in the months thereafter [4].

Secondly, the idea of understanding the feasibility of MD in a non-Mediterranean country has been a focus of researchers (ignored in Murphy and Parletta’s review [1]) for some time. We wish to highlight explicitly the work by Papadaki and colleagues [8–10], Logan et al. [11], Moore et al. [12], as well as the work of our team [5], which should have been considered in their article as they offer prominent and contemporary insight into the questions posed by the authors.

In conclusion, we share the authors’ excitement about the potential health benefits of implementing an MD in other non-accustomed cohorts such as an Australian cohort; particularly when additional cognitive and metal health outcomes are investigated. Nevertheless, the importance of acknowledging contribution is paramount for this field to move forward with intelligence and diligence to the factors which our health professionals, researchers and practitioner’s promoting the MD will face in broader community or research programmes. Therefore, a commentary was required to highlight the significant omissions and enhance the level of knowledge to readers of the journal on what has been achieved in this area. Our response has achieved this and we encourage reading of the cited references.
